# Supplementary material for: Endogenous opioids in the nucleus accumbens promote approach to high-fat food in the absence of caloric need
Source: eLife. 2018 Mar 27;7:e34955. doi: 10.7554/eLife.34955 (PMC5903865; doi:10.7554/eLife.34955)
Supplement: Transparent reporting form [file elife-34955-transrepform.docx]

***eLife’s* transparent reporting form**

We encourage authors to provide detailed information *within their submission* to facilitate the interpretation and replication of experiments. Authors can upload supporting documentation to indicate the use of appropriate reporting guidelines for health-related research (see [EQUATOR Network](http://www.equator-network.org/%20)), life science research (see the [BioSharing Information Resource](https://biosharing.org/)), or the [ARRIVE guidelines](http://www.plosbiology.org/article/info:doi/10.1371/journal.pbio.1000412) for reporting work involving animal research. Where applicable, authors should refer to any relevant reporting standards documents in this form.

If you have any questions, please consult our Journal Policies and/or contact us: [editorial@elifesciences.org](mailto:editorial@elifesciences.org).

**Sample-size estimation**

- You should state whether an appropriate sample size was computed when the study was being designed
- You should state the statistical method of sample size computation and any required assumptions
- If no explicit power analysis was used, you should describe how you decided what sample (replicate) size (number) to use

Please outline where this information can be found within the submission (e.g., sections or figure legends), or explain why this information doesn’t apply to your submission:

The sample sizes used in this study here are comparable to many previous studies featuring behavioral pharmacological and single-unit electrophysiological data.

**Replicates**

- You should report how often each experiment was performed
- You should include a definition of biological versus technical replication
- The data obtained should be provided and sufficient information should be provided to indicate the number of independent biological and/or technical replicates
- If you encountered any outliers, you should describe how these were handled
- Criteria for exclusion/inclusion of data should be clearly stated
- High-throughput sequence data should be uploaded before submission, with a private link for reviewers provided (these are available from both GEO and ArrayExpress)

Please outline where this information can be found within the submission (e.g., sections or figure legends), or explain why this information doesn’t apply to your submission:

For behavioral pharmacology experiments, the number of biological replicates (animals) used in each experiment can be found in the figure legend.

For electrophysiological recordings, the number of biological replicates (animals) and the number of technical replicates (behavioral sessions across which data were acquired) are reported in the figure legends. Numbers of neurons are reported on the relevant figure panels or in the text describing the result.

We identified 3 outlier neurons whose baseline firing rate was changed following drug injection. Statistical results are reported for an analysis in which outliers were removed, as well as an identical analysis in which they were not removed. Details can be found in the figure legend for Figure 5-figure supplement 2.

Criteria for inclusion of neural signals can be found in the Methods section (see *Analysis of neural data* subsection).

**Statistical reporting**

- Statistical analysis methods should be described and justified
- Raw data should be presented in figures whenever informative to do so (typically when N per group is less than 10)
- For each experiment, you should identify the statistical tests used, exact values of N, definitions of center, methods of multiple test correction, and dispersion and precision measures (e.g., mean, median, SD, SEM, confidence intervals; and, for the major substantive results, a measure of effect size (e.g., Pearson's r, Cohen's d)
- Report exact p-values wherever possible alongside the summary statistics and 95% confidence intervals. These should be reported for all key questions and not only when the p-value is less than 0.05.

Please outline where this information can be found within the submission (e.g., sections or figure legends), or explain why this information doesn’t apply to your submission:

Descriptions of all statistical tests, exact values of N, descriptions of center, and measures of precision can be found in either the figure legends or in the text describing the result. Detailed descriptions of procedures for Generalized Linear Modeling analysis can be found in the Methods section (see *Generalized Linear Model (GLM) fitting* subsection). When appropriate, p-values were adjusted for multiple comparisons; instances where such corrections occurred are indicated in the figure legends.

(For large datasets, or papers with a very large number of statistical tests, you may upload a single table file with tests, Ns, etc., with reference to sections in the manuscript.)

**Group allocation**

- Indicate how samples were allocated into experimental groups (in the case of clinical studies, please specify allocation to treatment method); if randomization was used, please also state if restricted randomization was applied
- Indicate if masking was used during group allocation, data collection and/or data analysis

Please outline where this information can be found within the submission (e.g., sections or figure legends), or explain why this information doesn’t apply to your submission:

As stated in the Methods (*Animals* subsection), animals were randomly assigned to their respective groups.

**Additional data files (“source data”)**

- We encourage you to upload relevant additional data files, such as numerical data that are represented as a graph in a figure, or as a summary table
- Where provided, these should be in the most useful format, and they can be uploaded as “Source data” files linked to a main figure or table
- Include model definition files including the full list of parameters used
- Include code used for data analysis (e.g., R, MatLab)
- Avoid stating that data files are “available upon request”

Please indicate the figures or tables for which source data files have been provided:

Custom scripts for the analysis of neural data were written in R and can be found here:

<https://github.com/kcaref/neural-analysis>

Source data will be made available upon request.
